# Supplementary material for: MultiCens: Multilayer network centrality measures to uncover molecular mediators of tissue-tissue communication
Source: PLoS Comput Biol. 2023 Apr 24;19(4):e1011022. doi: 10.1371/journal.pcbi.1011022 (PMC10159362; doi:10.1371/journal.pcbi.1011022)
Supplement: S1 Text — This document contains additional results on hormone-gene and hormone-lncRNA predictions, and AD vs. CTL networks using a different query set. This document also contains details of hyperparameters and method complexity. Additionally, it contains 5 supplemental tables (Table A-E in S1 Text) and 8 supplemental figures (Fig A-H in S1 Text), and pointers to two supplementary datasets (Data A-B in S1 Text). (PDF) [file pcbi.1011022.s001.pdf]

S1 Text: Supplementary Information for MultiCens (Multilayer network Centrality measures to uncover molecular mediators of tissue-tissue communication)

1 Supplementary Results . . . 1
1.1 Literature support for our hormone-gene predictions – additional information . . . 1
1.2 Literature support for our hormone-lncRNA predictions – additional information . . . 1
1.3 MultiCens analysis of AD vs. CTL networks using a different query set . . . 2
2 Supplementary Methods . . . 3
2.1 Hyperparameters and method complexity . . . 3
3 Supplementary Tables . . . 4
4 Supplementary Figures . . . 11
5 Supplementary Data/Files . . . 19

1 Supplementary Results

1.1 Literature support for our hormone-gene predictions – additional information

We discuss here the predictions for the growth hormone Somatotropin to supplement similar discussions in the Results section in the main text. To add to examples of novel predictions that are not in ground truth HGv1 and also have poor PubMed literature support scores, we discuss *S100A8* for Somatotropin – there is not substantial literature support for this prediction, but there are studies that show downregulation of *S100A8* on exogenous administration of the growth hormone [1]. *EGFR* (Epidermal growth factor receptor) playing key roles in development, cellular proliferation, and cancer, can be modulated by growth hormone [2,3]. Increased level of *FFAR4* (free fatty acid receptor 4) reduces ghrelin secretion, further stimulating hunger [4]. This context-based dependency is well captured in the cosine-based similarity in the embedding space, but the gene has low or no direct co-occurrence with hormone-related terms (see Fig. 4B of main text).

1.2 Literature support for our hormone-lncRNA predictions – additional information

We discuss here the long non-coding RNA (lncRNA) predictions for different hormones to supplement similar discussions in the Results section in the main text. Since Table C lists all supporting references for each hormone-lncRNA prediction, we do not cite all these references in the text below, similar to what we do in the main text for better readability.

We first discuss lncRNA predictions related to insulin. Our centrality-based ranking of insulin-relevant pancreas lncRNAs revealed *HOXA-AS2*, which has links to diabetes through another gene *TIMP3* [5].

Further, interplay between lncRNA and insulin pathway related genes are indicated in pathogenesis of different diseases [6]. This phenomenon is supported by our lncRNA prediction in pancreas, where most of them promote tumorigenesis and metastasis (*LINC00672*

promotes endometrial cancer chemosensitivity, *HOXA-AS2* promotes cellular processes aiding non-small cell lung cancer or pancreatic cancer, *PRR34-AS1* is highly expressed in hepatocellular carcinoma, and *LINC00294* induced by glucose-regulated protein 78, *GRP78*, aids in advancement of cervical cancer). Similarly, lncRNAs predicted to be present in skeletal muscle are also involved in tumorigenesis (*ZEB1-AS1* promotes pancreatic cancer progression, *TNK2-AS1*/miR-125a-5p fosters the progression of gastric cancer, whereas *PWAR6* and *PRRT3-AS1* act as tumour suppressors in glioma and prostate cancer respectively).

For the growth hormone Somatotropin, many predicted lncRNAs have connections to cancer cell growth as described next. While loss of *LINC01132* attenuates ovarian tumour growth, silencing lncRNA-*UCA1* leads to repression of pituitary cancer cell growth and prolactin (PRL) secretion and *LINC01473* expression is negatively correlated with serum interleukin-2 and tumor necrosis factor α levels in multiple myeloma.

Similarly, *PTPRD-AS1* is inversely correlated with the overall survival in patients with ovarian cancer. Further, *PTPRD* (receptor protein tyrosine phosphatase delta) hinders the growth of glioblastoma multiforme and other tumor cells, and also human astrocytes in dearth of *PTPRD* exhibits growth escalation. This probably indicates that *PTPRD-AS1*, by controlling PTPRD expression level, helps in glial cell growth.

LncRNAs found with high progesterone-specific query set centrality are involved in several cancers, including colon adenocarcinoma (*TAF1A-AS1*), prostate cancer (*PCAT19*), ovarian cancer (*HHIP-AS1*), breast cancer (*LINC00641*, *MIR210HG*, *HAGLR*), endometrial cancer (*MIR210HG*, *LINC01016*), and many others.

1.3 MultiCens analysis of AD vs. CTL networks using a different query set

We showed in main text how the change in the four-brain-region gene networks between Alzheimer's disease (AD) vs. Control (CTL) groups can be investigated by applying MultiCens with synaptic signaling genes (also referred to as synaptic genes or SSG) as the query set. When we changed the Synaptic signaling gene set (SSG, 134 genes) to Plaque-induced gene set (PIG, 57 genes) for the query set, MultiCens centralities of SSG vs. PIG were highly but not perfectly correlated (see Suppl Fig G). This resulted in top-ranking genes and pathway enrichments for PIG, some of which are similar between PIG and SSG as discussed first below, and others that are different. Brain region specific similarities and differences were also noted.

For instance, for PIG, we see that HSP90 chaperone cycle for steroid hormone receptors (SHR) pathway is again enriched in AD group in all 3 brain regions as for SSG. Similarly biological process "protein folding" is found to be enriched in both PIG and SSG set in AD group. Moreover, *JMJD6*, *SLC5A3*, *CIRBP*, and *AHSA1* are also among the top ten genes in AD group (as for SSG; see Results in main text for SSG top-ranking genes). Pathway related to extracellular matrix (ECM) organization (R-HSA-1474244) is also enriched for correlation to PIG genes in AD brain regions. The ECM is known to contribute to both Aβ plaques' formation and degradation [7]. In case of control group, pathway related to immune system and biological process concerning cytokine production are positively enriched. Aβ is a known constituent of the innate immune system and regarded as an "early responder cytokine" [8]. The change in gene ranking and pathway enrichments for top ranks for PIG relative to SSG is highlighted in Suppl Fig. Ha and Hb respectively (compare with Fig. 5 of main text). For instance, biological processes like "regulation of hemopoiesis" and pathways like "Serotonin Neurotransmitter Release Cycle", "ER to Golgi Anterograde Transport" and "Interleukin-4 and Interleukin-13 signaling" were found in PIG-based, but not SSG-based,

enrichment analysis. On the other hand, pathways like "Voltage gated Potassium channels", "Cell-cell junction organization" and "The role of GTSE1 in G2/M progression after G2 checkpoint" and biological processes like "axon development" were prominently enriched for SSG gene set.

2 Supplementary Methods

2.1 Hyperparameters and method complexity

Following the conventions of PageRank centrality algorithm, we tested p in the range of $[0.7, 0.95]$. The higher values of p tend to assign higher centrality scores to genes that are part of communities as compared to smaller values of p. In all our experiments, we report results at $p = 0.9$. However, there is very small deviation of rankings between $p = 0.85$ (typical value of p used for web-based networks) and $p = 0.9$.

In the proposed method, the following two steps are involved:

1. Construction of multilayer network
2. Centrality score computation

The first step of network construction can be performed using multiple ways. In this project, we opted for correlation-based methods; hence we calculate correlation for all possible gene-gene pairs. Assuming a multilayer network with L layers and n genes per layer, we need to compute the correlation between $(L \times n)^2$ pairs. Each correlation can be computed with $O(k)$ time complexity, where k is the number of samples. So the total runtime complexity of network construction is $O(k(L \times n)^2)$. In this project, we worked predominantly with on two-layered networks with around 15k genes per layer. The number of samples can be in few hundreds depending upon the intersection of sample IDs between the tissues. In our experiments, it takes roughly three hours to generate a two-layered multilayer network. The network can be stored in an adjacency matrix or list format. We store these networks in an adjacency matrix format of size $(L \times n)^2$. In practice, the matrix file can take up to a few GBs of memory.

The second step, which is the major contribution of work - the centrality computation, uses iterative equations to find the scores. Each iteration takes $O(nL \times nL)$ computations. The number of iterations depends upon various factors such as the diameter of the graph, modularity of the graph, etc. In practice, the method converges under 50 iterations incurring a total time of around thirty minutes. This step can be made efficient by distributing the code to multiple machines similar to distributed PageRank [9].

3 Supplementary Tables

S. No.	Hormone	Gene-set (size)	AUC (coexp)	AUC (coexp + SNAP)
1	Adrenaline	Target (24)	0.469	0.494
2	Aldosterone	Source (11)	0.458	0.458
3	Angiotensin	Target (15)	0.507	0.557
4	Cortisol	Source (12)	0.533	0.542
5	Estradiol	Target (89)	0.493	0.512
6	Glucagon	Target (19)	0.552	0.574
7	Insulin	Source (156)	0.668	0.677
8	Insulin	Target (215)	0.664	0.685
9	Norepinephrine	Source (16)	0.473	0.475
10	Norepinephrine	Target (14)	0.430	0.471
11	Progesterone	Source (13)	0.740	0.733
12	Progesterone	Target (35)	0.598	0.645
13	Somatotropin	Source (10)	0.679	0.712
14	Somatotropin	Target (22)	0.564	0.671
15	Thyroxin	Source (12)	0.482	0.498
16	Vitamin-D	Target (41)	0.570	0.578

Table A: Area under recall-at-k curve (AUC) for the ranking obtained using MultiCens query-set centralities, which were computed in the hormone-related human multilayer networks' application. For comparison, AUC for a random ranking of all genes is 0.5. We evaluated only hormones with at least 10 genes on the source or target tissue side, so that these gene sets to be retrieved are sufficiently large to yield a reliable estimate of AUC (see Suppl Fig B and Fig 4A of main text for recall-at-k curves; see also Fig 4B (Coexpression+SNAP based results) of main text for more information about this application/evaluation, and a visualization of this table).

(a) Insulin: Pancreas (predictions of insulin-producing genes)	
Top genes	Gene names
SYBU	syntabulin
LRP1	LDL receptor related protein 1
CNR1	cannabinoid receptor 1
PTPRN2	protein tyrosine phosphatase receptor type N2
SERP1	stress associated endoplasmic reticulum protein 1
CD74	CD74 molecule
LRRC8A	leucine rich repeat containing 8 VRAC subunit A
PICK1	protein interacting with PRKCA 1
EGFR	epidermal growth factor receptor
INS	insulin

(b) Somatotropin: Pituitary gland (predictions of somatotropin-producing genes)	
Top genes	Gene names
HDAC1	histone deacetylase 1
EGFR	epidermal growth factor receptor
FKBP1B	FKBP prolyl isomerase 1B
FFAR4	free fatty acid receptor 4
S100A8	S100 calcium binding protein A8
PER2	period circadian regulator 2
RFX3	regulatory factor X3
PRKCE	protein kinase C epsilon
SERP1	stress associated endoplasmic reticulum protein 1
ITSN1	intersectin 1

Table B: Gene names of the top 10 predicted genes by MultiCens (ranked only among genes involved in peptide secretion) for the two primary peptide hormones: (a) insulin, and (b) somatotropin. See also Fig 4B in main text for more context.

	Insulin			
	Pancreas		Skeletal Muscle	
	lncRNA symbol	References	lncRNA symbol	References
1	LINC00672	[10,11]	ZEB1-AS1	[12,13,14,15]
2	HOXA-AS2	[5,16,17]	TNK2-AS1	[18]
3	PRR34-AS1	[19,20]	PWAR6	[19,21,22,23]
4	MIR22HG	[24]	PRRT3-AS1	[25,26]
5	LINC00294	[27]	PRKCQ-AS1	[28,29]

	Somatotropin			
	Pituitary Gland		Liver	
	lncRNA symbol	References	lncRNA symbol	References
1	LINC01588	[30]	NEAT1	[31]
2	PTPRD-AS1	[32,33]	ZNF528-AS1	[34]
3	LINC01132	[35]	MIR210HG	[36,37]
4	UCA1	[38]	ALMS1-IT1	None
5	LINC01473	[39]	LINC01278	[40]

	Progesterone			
	Ovaries		Uterus	
	lncRNA symbol	References	lncRNA symbol	References
1	CCDC18-AS1	None	HAGLR	[41,42]
2	LINC00641	[43,44,45]	TAF1A-AS1	[46]
3	MIR210HG	[47,48,49,50]	LINC00602	None
4	LINC01016	[51,52]	PCAT19	[53]
5	BEAN1-AS1	None	HHIP-AS1	[54]

	Norepinephrine			
	Adrenal Glands		Small Intestine	
	lncRNA symbol	References	lncRNA symbol	References
1	PGM5P4-AS1	None	RNF139-AS1	None
2	CCDC18-AS1	None	CARMN	[55]
3	MAGI2-AS3	[56]	SPATA41	[57]
4	LINC01291	None	GHET1	[58]
5	TOLLIP-AS1	None	ATP1B3-AS1	None

Table C: Top predicted lncRNAs for the hormones along with the references. These references show association of these lncRNAs to the corresponding hormone and related diseases.

Gene Set	Description	Size	Leading Edge Number	ES	NES	P Value	FDR	Region
**Gene Ontology Biological Process**								
GO:0008380	RNA splicing	28	16	-0.6329	-2.0867	<2.2e-16	0.01574	BM10
GO:0055067	monovalent inorganic cation homeostasis	45	21	0.5482	1.9762	<2.2e-16	0.034546	BM44
GO:0002526	acute inflammatory response	70	29	0.52706	2.0248	<2.2e-16	0.036662	BM44
GO:0045927	positive regulation of growth	63	24	0.50935	1.9251	<2.2e-16	0.047002	BM44
**Reactome Pathways**								
R-HSA-6783783	Interleukin-10 signaling	26	20	0.65246	2.0994	<2.2e-16	0.0083675	BM10
R-HSA-446652	Interleukin-1 family signaling	24	11	0.60961	1.9713	<2.2e-16	0.04393	BM10
R-HSA-168142	Toll Like Receptor 10 (TLR10) Cascade	14	7	0.7634	2.1006	<2.2e-16	0.0021587	BM36
R-HSA-168176	Toll Like Receptor 5 (TLR5) Cascade	14	7	0.7634	2.1006	<2.2e-16	0.0021587	BM36
R-HSA-975871	MyD88 cascade initiated on plasma membrane	14	7	0.7634	2.1006	<2.2e-16	0.0021587	BM36
R-HSA-168898	Toll-like Receptor Cascades	43	24	0.58042	2.0905	<2.2e-16	0.0021987	BM36
R-HSA-5660526	Response to metal ions	8	8	0.93365	2.1595	<2.2e-16	0.0023985	BM36
R-HSA-5661231	Metallothioneins bind metals	8	8	0.93365	2.1595	<2.2e-16	0.0023985	BM36
R-HSA-168179	Toll Like Receptor TLR1:TLR2 Cascade	23	15	0.62753	1.9481	<2.2e-16	0.020987	BM36
R-HSA-181438	Toll Like Receptor 2 (TLR2) Cascade	23	15	0.62753	1.9481	<2.2e-16	0.020987	BM36
R-HSA-168249	Innate Immune System	277	117	0.39952	1.9139	<2.2e-16	0.027583	BM36

Table D: Biological Process and Pathways resulting from delta rank generated from subtracting global centrality rank from local centrality rank.

Gene Set	Description	Size	Leading Edge Number	ES	NES	P Value	FDR	Region
Gene Ontology Biological Process								
GO:0099565	chemical synaptic transmission, postsynaptic	42	18	0.919324949	2.195971926	0	0	BM36
GO:0099177	regulation of trans-synaptic signaling	112	39	0.848760689	2.195839339	0	0	BM36
GO:0007218	neuropeptide signaling pathway	55	9	0.879497989	2.149822296	0	0	BM36
GO:0031644	regulation of neurological system process	52	19	0.87180967	2.147292714	0	0	BM36
GO:0097305	response to alcohol	81	11	0.846794752	2.128140833	0	2.18E-04	BM36
GO:0042391	regulation of membrane potential	138	28	0.81126512	2.110264992	0	5.44E-04	BM36
GO:0050890	cognition	79	23	0.827294259	2.091058236	0	0.001554	BM36
GO:0043270	positive regulation of ion transport	107	26	0.81078917	2.069562108	0	0.002770	BM36
GO:0034764	positive regulation of transmembrane transport	58	12	0.839063719	2.073512618	0	0.002829	BM36
GO:0006836	neurotransmitter transport	86	25	0.823336238	2.073846582	0	0.003023	BM36
GO:0046677	response to antibiotic	106	12	0.803376055	2.075485818	0	0.003265	BM36
GO:0001505	regulation of neurotransmitter levels	107	24	0.799179839	2.057119521	0	0.00399	BM36
GO:0032409	regulation of transporter activity	78	24	0.803096338	2.03929486	0	0.005121	BM36
GO:0030534	adult behavior	35	10	0.875698896	2.042144761	0	0.005305	BM36
GO:0007215	glutamate receptor signaling pathway	25	13	0.899482511	2.042296528	0.001402	0.005586	BM36
GO:0007271	synaptic transmission, cholinergic	16	5	0.945045923	2.047100423	0	0.005776	BM36
GO:0050795	regulation of behavior	29	3	0.883577239	2.043435984	0	0.005830	BM36
GO:0007274	neuromuscular synaptic transmission	14	7	0.940214749	2.030576104	0	0.006409	BM36
GO:0050808	synapse organization	85	24	0.792245141	2.02016962	0	0.008706	BM36
GO:0030902	hindbrain development	37	10	0.823300636	2.013836426	0.001287	0.009224	BM36
GO:0007626	locomotory behavior	46	7	0.843479789	2.015323452	0	0.009305	BM36
GO:0015837	amine transport	41	9	0.821377158	2.007656345	0.001269	0.010091	BM36
GO:0009636	response to toxic substance	177	17	0.762777266	1.999890796	0	0.012018	BM36
GO:0060359	response to ammonium ion	46	13	0.832336121	1.997402596	0	0.012107	BM36
GO:1990778	protein localization to cell periphery	56	14	0.813860088	1.989762943	0.001187	0.012309	BM36
GO:1902115	regulation of organelle assembly	25	5	0.848104021	1.991872093	0.004310	0.012495	BM36
GO:0034765	regulation of ion transmembrane transport	156	39	0.765984009	1.9902445	0	0.012671	BM36

GO:0017156	calcium ion regulated exocytosis	41	16	0.812372672	1.992276495	0.003816	0.012892	BM36
GO:0072347	response to anesthetic	29	10	0.858780703	1.993538619	0	0.013016	BM36
GO:0043279	response to alkaloid	37	7	0.837396975	1.977086053	0.001317	0.016796	BM36
GO:0050905	neuromuscular process	22	6	0.866247981	1.966512983	0	0.017060	BM36
GO:0051648	vesicle localization	50	24	0.786291276	1.966575067	0.001236	0.017504	BM36
GO:0031346	positive regulation of cell projection organization	62	18	0.785039946	1.968085679	0	0.017631	BM36
GO:0099504	synaptic vesicle cycle	40	15	0.818579662	1.969306562	0.001287	0.017765	BM36
GO:0035249	synaptic transmission, glutamatergic	29	10	0.863998243	1.970157371	0	0.017974	BM36
GO:0098742	cell-cell adhesion via plasma-membrane adhesion molecules	80	18	0.765585418	1.970620217	0	0.018331	BM36
GO:0099601	regulation of neurotransmitter receptor activity	22	10	0.873835712	1.970719371	0.00284	0.018782	BM36
GO:0007631	feeding behavior	38	6	0.816911274	1.954524562	0.001290	0.022110	BM36
GO:0099003	vesicle-mediated transport in synapse	39	15	0.812712348	1.952143504	0.001278	0.022520	BM36
GO:0007187	G protein-coupled receptor signaling pathway, coupled to cyclic nucleotide second messenger	104	24	0.765355958	1.943876184	0	0.026092	BM36
GO:0007200	phospholipase C-activating G protein-coupled receptor signaling pathway	54	6	0.782911885	1.941841206	0.001242	0.026332	BM36
GO:0043627	response to estrogen	29	2	0.828315929	1.940002762	0.005457	0.027001	BM36
GO:0015844	monoamine transport	40	9	0.800824105	1.935027859	0	0.029258	BM36
GO:0010975	regulation of neuron projection development	92	27	0.75769345	1.929702021	0	0.031053	BM36
GO:0023061	signal release	129	24	0.75047805	1.930681609	0	0.031190	BM36
GO:0050803	regulation of synapse structure or activity	56	19	0.779498741	1.922232925	0.001210	0.034637	BM36
GO:0009410	response to xenobiotic stimulus	98	10	0.749804746	1.916566497	0.001086	0.035189	BM36
GO:0009308	amine metabolic process	32	2	0.817892028	1.918110168	0	0.03538	BM36
GO:0072511	divalent inorganic cation transport	152	29	0.740238571	1.919887555	0	0.035382	BM36
GO:0033500	carbohydrate homeostasis	59	8	0.786442314	1.916993761	0.001175	0.035675	BM36
GO:0060560	developmental growth involved in morphogenesis	55	19	0.790382904	1.918387113	0	0.035960	BM36
GO:0010959	regulation of metal ion transport	125	22	0.747349351	1.914196868	0	0.036166	BM36
GO:0019233	sensory perception of pain	41	7	0.790721052	1.912264889	0.003851	0.036551	BM36
GO:0009743	response to carbohydrate	58	9	0.776617549	1.911212912	0.001168	0.036620	BM36

GO:2001023	regulation of response to drug	34	6	0.78993049	1.908633729	0.010498	0.037953	BM36
GO:0033555	multicellular organismal response to stress	36	7	0.798770892	1.905217786	0.003856	0.039724	BM36
GO:0035418	protein localization to synapse	13	5	0.892149701	1.903371202	0.007936	0.040268	BM36
GO:0007272	ensheathment of neurons	27	4	0.828563031	1.902352519	0.006839	0.040305	BM36
GO:0007586	digestion	49	6	0.758897165	1.899424799	0.002415	0.041320	BM36
GO:0050886	endocrine process	36	5	0.806448881	1.899914694	0.003861	0.041559	BM36
GO:0071241	cellular response to inorganic substance	63	14	0.775512987	1.895405407	0	0.043479	BM36
**Reactome**								
R-HSA-112316	Neuronal System	109	43	0.87027	2.1774	<2.2e-16	<2.2e-16	BM36
R-HSA-112315	Transmission across Chemical Synapses	62	26	0.88727	2.1721	<2.2e-16	<2.2e-16	BM36
R-HSA-112314	Neurotransmitter receptors and postsynaptic signal transmission	44	20	0.89028	2.1123	<2.2e-16	0.00170	BM36

Table E: Biological Process and Pathways resulting from delta rank generated from subtracting query-set centrality rank from global centrality rank

4 Supplementary Figures

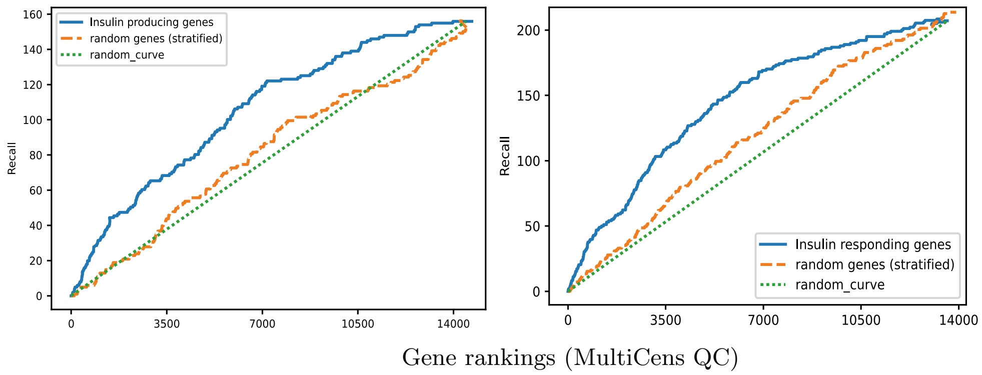

Fig. A: Comparison of gene rankings of ground truth sets (hormone-producing and responding) with a random gene set chosen by stratification on the gene variance level. Blue curve corresponds to the actual ground truth gene set, orange curve represents the ranking obtained by a random set of genes chosen by stratifying over gene variance levels, and green curve is the average curve obtained using any random set of genes. Note that the random gene sets we consider have the same size as the actual ground truth set.

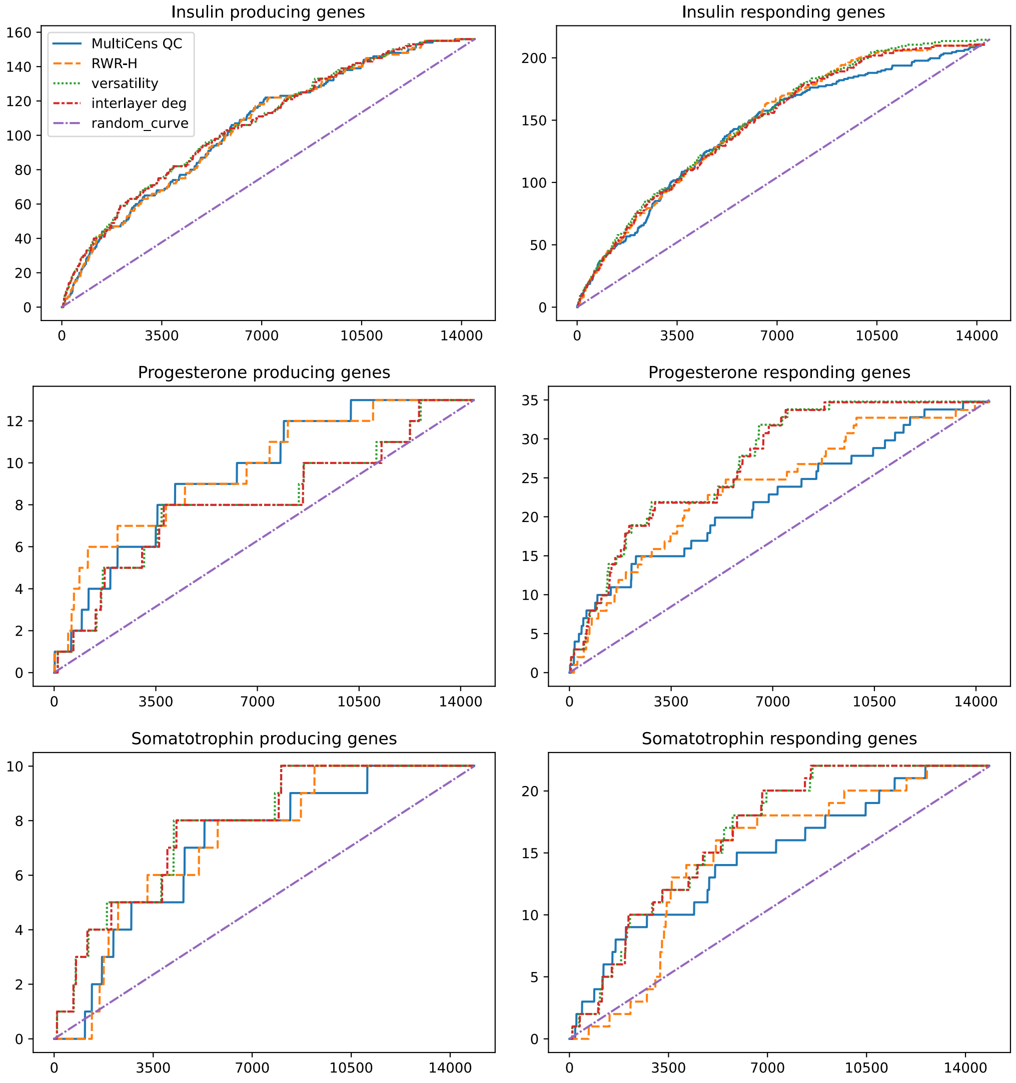

Fig. B: Recall (number of ground truth genes recovered; y-axis) in the top k ranked genes (x-axis) are plotted using MultiCens query-set centrality (MultiCens QC) based ranking vis-'a-vis rankings obtained by baseline methods.

Hormone Name	Number of Source Genes	Number of Target Genes	Retrieving Source genes	Retrieving Target genes
Aldosterone	11	2	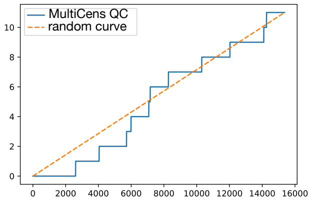	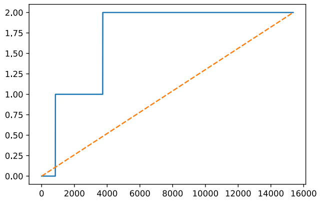
Angiotensin-IV	1	15	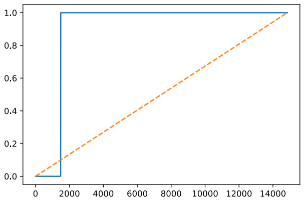	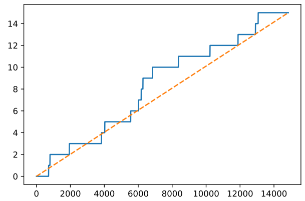
Adrenaline	4	24	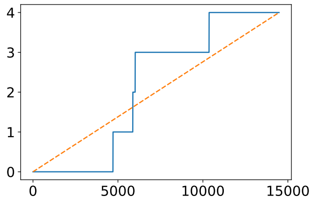	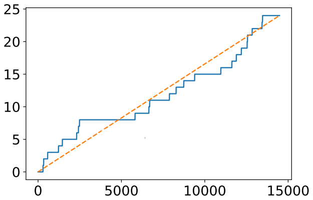
Cortisol	12	3	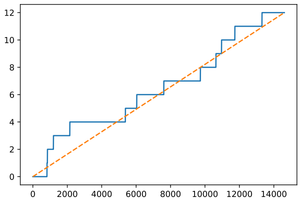	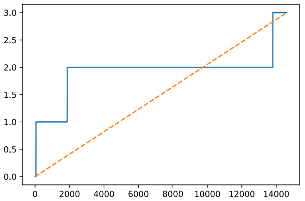
Estradiol	2	89	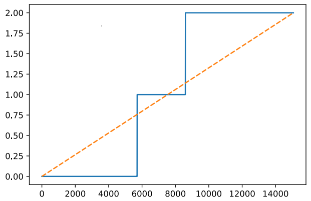	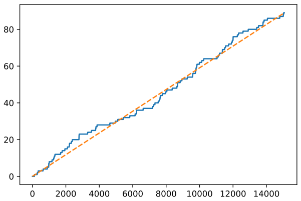
Glucagon	7	19	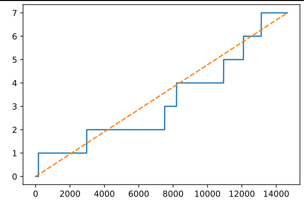	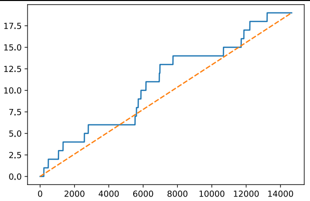

Hormone Name	Number of Source Genes	Number of Target Genes	Retrieving Source genes	Retrieving Target genes
Thyroxin	12	8	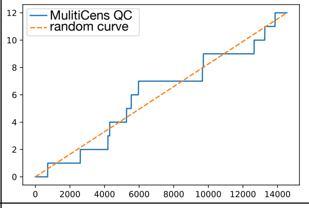	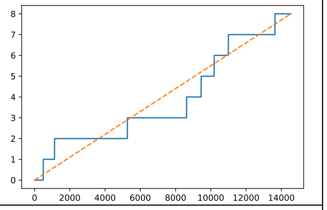
Vitamin-D	8	41	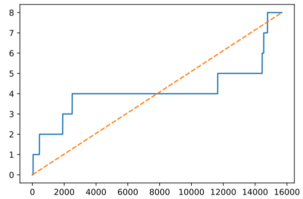	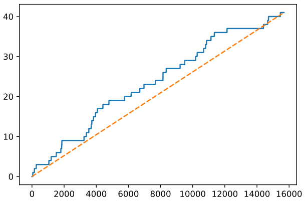

Fig. C: Performance of MultiCens on all tested non-primary hormones, i.e., hormones with insufficient gene associations in the ground-truth in the following sense – these hormones have at least 10 genes in either the producing (source) set or the responding (target) set, but *not* in both sets unlike the primary hormones. See Fig 4A of main text for similar recall-at-k curves for the primary hormones; Fig 4B of the main text and Table A summarize the area under these curves for all tested hormones.

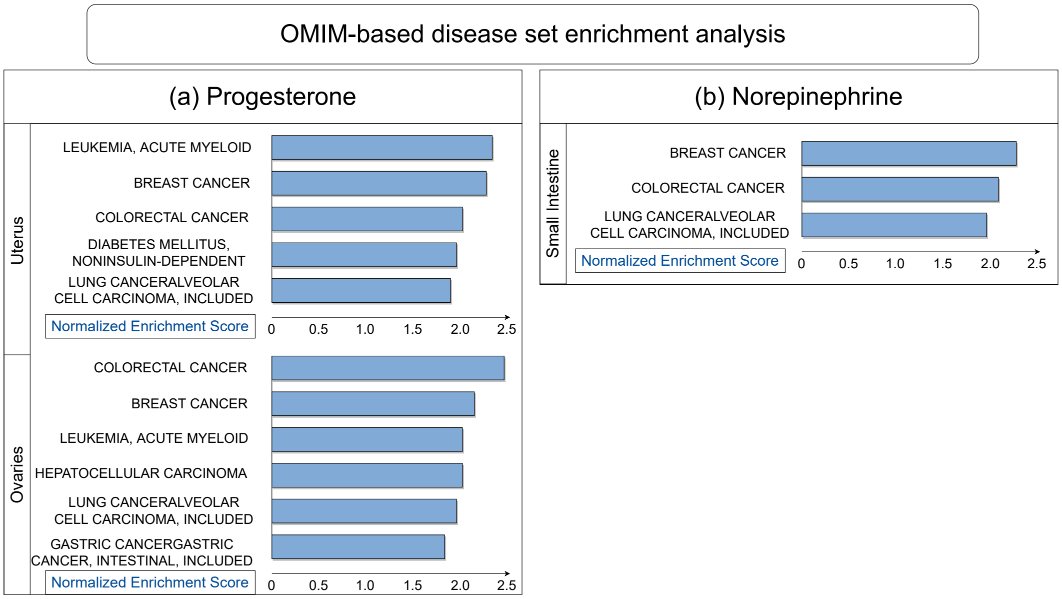

Fig. D: OMIM-based disease set enrichment analysis of the centrality scores. We use WebGestalt to get these enrichments and apply an FDR cut-off of 0.05. For Norepinephrine, we do not see any significant enrichments at this FDR cutoff in Adrenal Glands. See also Fig 4A in main text for similar enrichment analysis for the other two primary hormones: insulin and somatotropin.

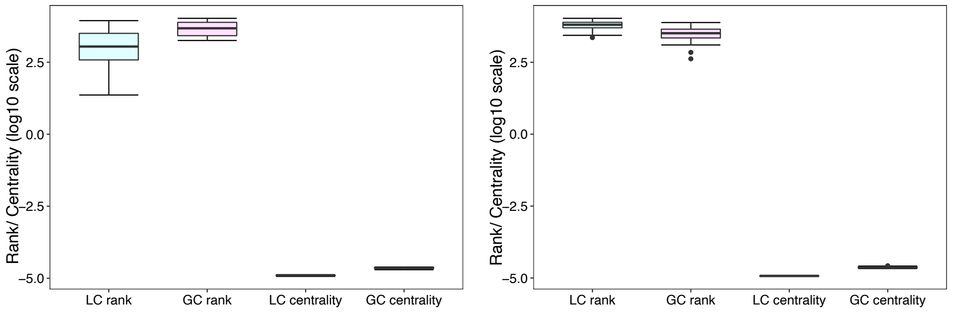

(a) RNA splicing (b) Acute inflammatory response

Fig. E: Different ranks and centrality scores of the genes (y-axis in log-scale), participating in enriched biological process resulting from LC-GC delta rank, are highlighted in the box plots. While RNA splicing seems to be predominant (influential) in the intra-region gene network, Acute inflammatory response seems to be influential in the inter-region gene network due to its better global centrality ranks.

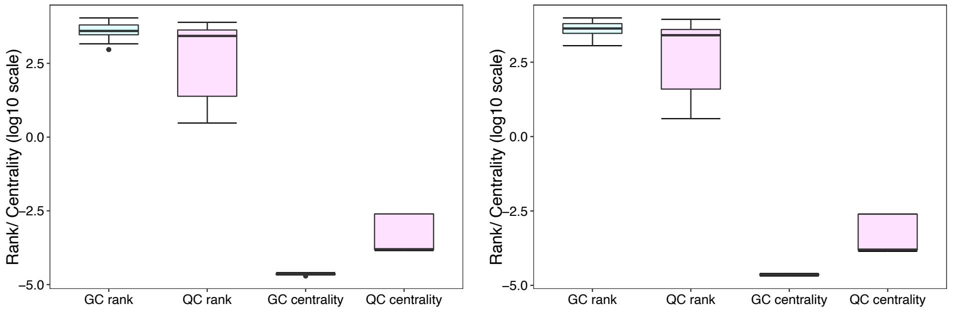

(a) Neuronal System (b) Regulation of trans-synaptic signaling

Fig. F: Different ranks and centrality scores of the genes (y-axis in log-scale), participating in enriched pathways resulting from GC-QC delta rank, are highlighted in the box plots. Both neuronal system pathway and trans-synaptic signaling regulation are better connected to the query-set (synaptic signaling genes) as expected.

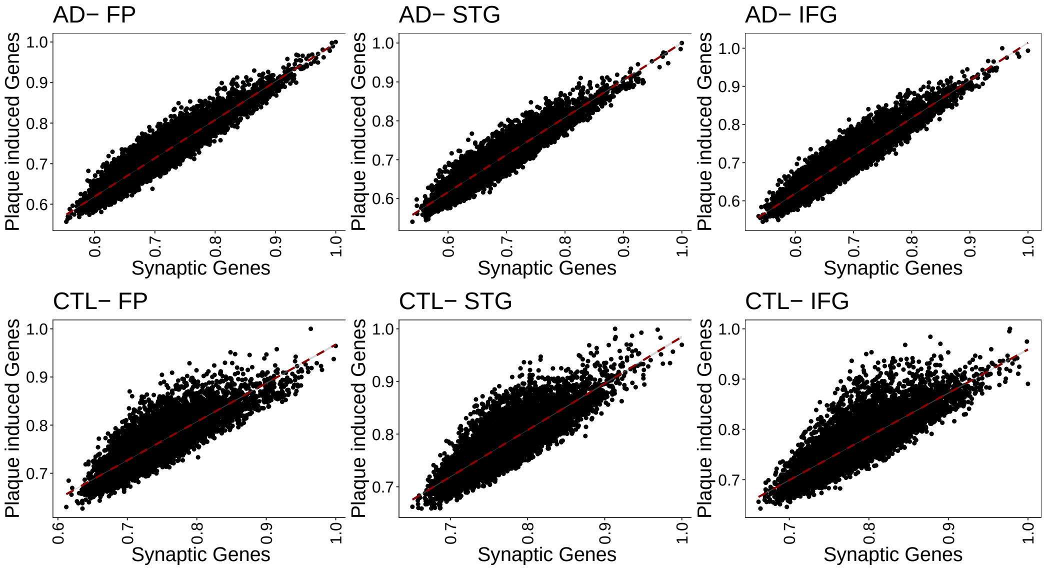

Fig. G: Scatter plots representing correlation of centrality scores obtained using SSG vs PIG-based query sets. It can be inferred that the centrality scores with respect to different query sets shows less deviation in AD-based multilayer network than the control group.

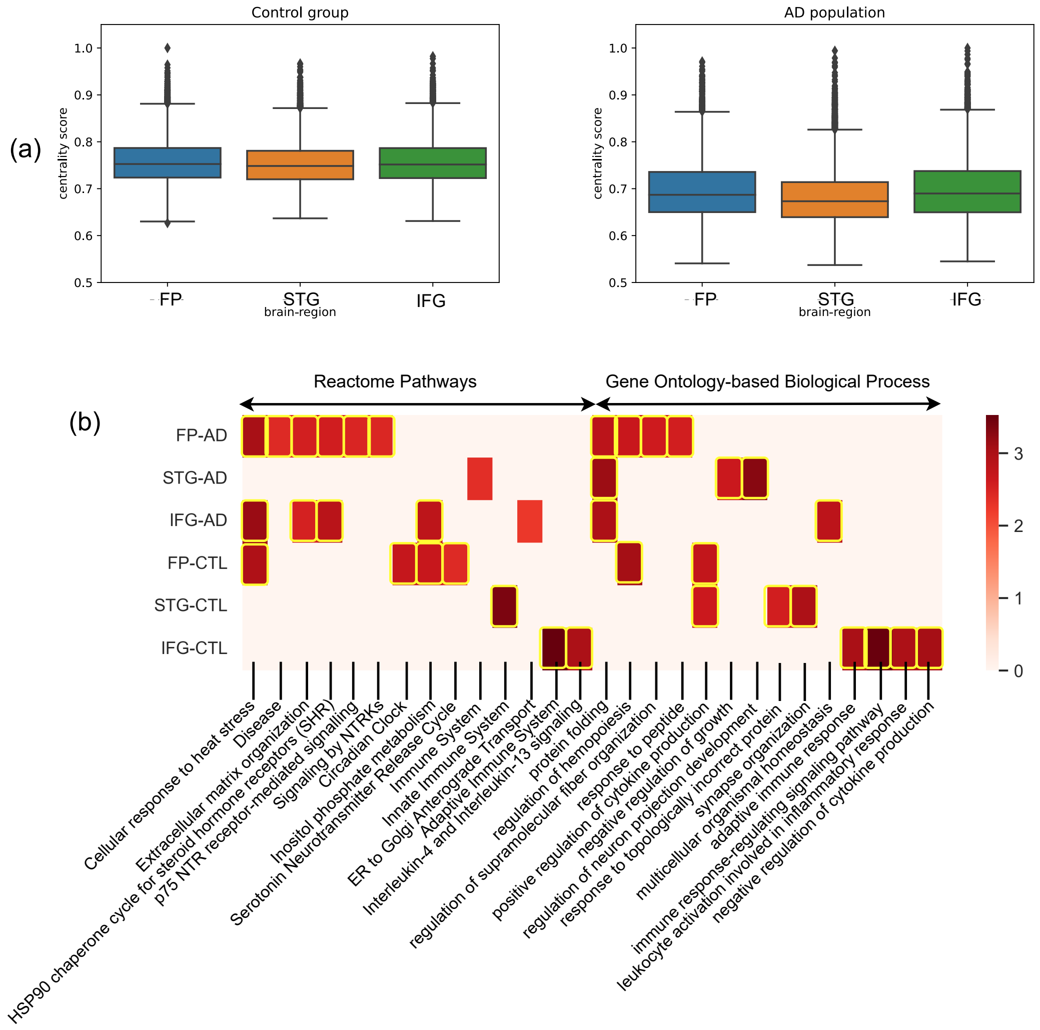

Fig. H: **PIG-based query set**: Study of changes in the centrality-based gene rankings of four-layer networks of control and Alzheimer affected population. The PIG *query-set* is present in parahippocampal gyrus (PHG) and we rank genes of frontal pole (FP), superior temporal gyrus (STG) and inferior frontal gyrus (IFG). (a) Bar-plot showing region-wise shift of centrality scores of the three regions. (b) Reactome pathways and Gene Ontology-based process (GO-BP) enrichment analysis of each region in control and AD state. Color map represents the normalized enrichment score from WebGestalt. The highlighted boxes pass the 0.01 FDR cut-off. If centrality-based gene rankings of a region do not pass the 0.05 FDR cut off for an enrichment, we set the corresponding normalized enrichment score to 0.

5 Supplementary Data/Files

Data A: MultiCens on human multilayer networks, related to the four primary hormones. Link: https://github.com/BIRDSgroup/MultiCens/tree/main/results.

Data B: Results using brain region networks for AD and CTL populations. Link: https://github.com/BIRDSgroup/MultiCens/tree/main/brain_region_results.

References

1. L. Chung, A. E. Nelson, K. K. Ho, and R. C. Baxter, "Proteomic profiling of growth hormone-responsive proteins in human peripheral blood leukocytes," *J Clin Endocrinol Metab*, vol. 94, pp. 3038–3043, Aug 2009.
2. L. González, J. G. Miquet, P. E. Irene, M. E. Díaz, S. P. Rossi, A. I. Sotelo, M. B. Frungieri, C. M. Hill, A. Bartke, and D. Turyn, "Attenuation of epidermal growth factor (EGF) signaling by growth hormone (GH)," *J Endocrinol*, vol. 233, pp. 175–186, 05 2017.
3. E. Kostopoulou, A. P. Rojas-Gil, A. Karvela, and B. E. Spiliotis, "Epidermal growth factor receptor (EGFR) involvement in successful growth hormone (GH) signaling in GH transduction defect," *J Pediatr Endocrinol Metab*, vol. 30, pp. 221–230, Feb 2017.
4. D. Y. Oh and E. Walenta, "Omega-3 Fatty Acids and FFAR4," *Front Endocrinol (Lausanne)*, vol. 5, p. 115, 2014.
5. X. Li and H. M. Yu, "Overexpression of HOXA-AS2 inhibits inflammation and apoptosis in podocytes via sponging miRNA-302b-3p to upregulate TIMP3," *Eur Rev Med Pharmacol Sci*, vol. 24, pp. 4963–4970, 05 2020.
6. C. Dieter, N. E. Lemos, N. R. F. Corrêa, T. S. Assmann, and D. Crispim, "The Impact of lncRNAs in Diabetes Mellitus: A Systematic Review and In Silico Analyses," *Front Endocrinol (Lausanne)*, vol. 12, p. 602597, 2021.
7. Y. Sun, S. Xu, M. Jiang, X. Liu, L. Yang, Z. Bai, and Q. Yang, "Role of the Extracellular Matrix in Alzheimer's Disease," *Front Aging Neurosci*, vol. 13, p. 707466, 2021.
8. D. F. Weaver, "Amyloid beta is an early responder cytokine and immunopeptide of the innate immune system," *Alzheimers Dement (N Y)*, vol. 6, no. 1, p. e12100, 2020.
9. A. D. Sarma, A. R. Molla, G. Pandurangan, and E. Upfal, "Fast distributed pagerank computation," in *International Conference on Distributed Computing and Networking*, pp. 11–26, Springer, 2013.
10. W. Li, H. Li, L. Zhang, M. Hu, F. Li, J. Deng, M. An, S. Wu, R. Ma, J. Lu, *et al.*, "Long non-coding rna linc00672 contributes to p53 protein-mediated gene suppression and promotes endometrial cancer chemosensitivity," *Journal of Biological Chemistry*, vol. 292, no. 14, pp. 5801–5813, 2017.
11. D. Li, J. Lu, H. Li, S. Qi, and L. Yu, "Identification of a long noncoding rna signature to predict outcomes of glioblastoma," *Molecular medicine reports*, vol. 19, no. 6, pp. 5406–5416, 2019.
12. L. Gu, H. Sun, and Z. Yan, "lncrna zeb1-as1 is downregulated in diabetic lung and regulates lung cell apoptosis," *Experimental and Therapeutic Medicine*, vol. 20, no. 6, pp. 1–1, 2020.
13. Q. Meng, X. Zhai, Y. Yuan, Q. Ji, and P. Zhang, "lncrna zeb1-as1 inhibits high glucose-induced emt and fibrogenesis by regulating the mir-216a-5p/bmp7 axis in diabetic nephropathy," *Brazilian Journal of Medical and Biological Research*, vol. 53, no. 4, 2020.
14. Y. Song, C. Miao, and J. Wang, "Lncrna zeb1-as1 inhibits renal fibrosis in diabetic nephropathy by regulating the mir-217/mafb axis," *RSC advances*, vol. 9, no. 52, pp. 30389–30397, 2019.
15. G. Wei, T. Lu, J. Shen, and J. Wang, "LncRNA ZEB1-AS1 promotes pancreatic cancer progression by regulating miR-505-3p/TRIB2 axis," *Biochem Biophys Res Commun*, vol. 528, pp. 644–649, 08 2020.
16. Y. Lian, Z. Li, Y. Fan, Q. Huang, J. Chen, W. Liu, C. Xiao, and H. Xu, "The lncRNA-HOXA-AS2/EZH2/LSD1 oncogene complex promotes cell proliferation in pancreatic cancer," *Am J Transl Res*, vol. 9, no. 12, pp. 5496–5506, 2017.
17. F. X. Zheng, X. Q. Wang, W. X. Zheng, and J. Zhao, "Long noncoding RNA HOXA-AS2 promotes cell migration and invasion via upregulating IGF-2 in non-small cell lung cancer as an oncogene," *Eur Rev Med Pharmacol Sci*, vol. 23, pp. 4793–4799, Jun 2019.
18. L. Guo, H. Ma, Y. Kong, G. Leng, G. Liu, and Y. Zhang, "Long non-coding RNA TNK2 AS1/microRNA-125a-5p axis promotes tumor growth and modulated phosphatidylinositol 3 kinase/AKT pathway," *J Gastroenterol Hepatol*, vol. 37, pp. 124–133, Jan 2022.
19. G.-M. Liu, H.-D. Zeng, C.-Y. Zhang, and J.-W. Xu, "Key genes associated with diabetes mellitus and hepatocellular carcinoma," *Pathology-Research and Practice*, vol. 215, no. 11, p. 152510, 2019.

20. Z. Liu, Z. Li, B. Xu, H. Yao, S. Qi, and J. Tai, "Long Noncoding RNA PRR34-AS1 Aggravates the Progression of Hepatocellular Carcinoma by Adsorbing microRNA-498 and Thereby Upregulating FOXO3," *Cancer Manag Res*, vol. 12, pp. 10749–10762, 2020.
21. T. Nagai and M. Mori, "Prader-willi syndrome, diabetes mellitus and hypogonadism," *Biomedicine & pharmacotherapy*, vol. 53, no. 10, pp. 452–454, 1999.
22. R. Basheer, M. J. A. Jalal, and R. Gomez, "An unusual case of adolescent type 2 diabetes mellitus: Prader–willi syndrome," *Journal of family medicine and primary care*, vol. 5, no. 1, p. 181, 2016.
23. C. Zhang, H. Liu, P. Xu, Y. Tan, Y. Xu, L. Wang, B. Liu, Q. Chen, and D. Tian, "Identification and validation of a five-lncRNA prognostic signature related to Glioma using bioinformatics analysis," *BMC Cancer*, vol. 21, p. 251, Mar 2021.
24. Z. Lin, X. Li, X. Zhan, L. Sun, J. Gao, Y. Cao, and H. Qiu, "Construction of competitive endogenous rna network reveals regulatory role of long non-coding rnas in type 2 diabetes mellitus," *Journal of cellular and molecular medicine*, vol. 21, no. 12, pp. 3204–3213, 2017.
25. S. Yang, Y. Zhou, X. Zhang, L. Wang, J. Fu, X. Zhao, and L. Yang, "The prognostic value of an autophagy-related lncrna signature in hepatocellular carcinoma," *BMC bioinformatics*, vol. 22, no. 1, pp. 1–16, 2021.
26. L. Fan, H. Li, and W. Wang, "Long non-coding RNA PRRT3-AS1 silencing inhibits prostate cancer cell proliferation and promotes apoptosis and autophagy," *Exp Physiol*, vol. 105, pp. 793–808, 05 2020.
27. J. Qiu, S. Zhou, W. Cheng, and C. Luo, "LINC00294 induced by GRP78 promotes cervical cancer development by promoting cell cycle transition," *Oncol Lett*, vol. 20, p. 262, Nov 2020.
28. J. A. Timmons, P. J. Atherton, O. Larsson, S. Sood, I. O. Blokhin, R. J. Brogan, C.-H. Volmar, A. R. Josse, C. Slentz, C. Wahlestedt, *et al.*, "A coding and non-coding transcriptomic perspective on the genomics of human metabolic disease," *Nucleic acids research*, vol. 46, no. 15, pp. 7772–7792, 2018.
29. S. I. Itani, W. J. Pories, K. G. Macdonald, and G. L. Dohm, "Increased protein kinase C theta in skeletal muscle of diabetic patients," *Metabolism*, vol. 50, pp. 553–557, May 2001.
30. J. Volejnikova, P. Vojta, H. Urbankova, R. Mojzíkova, M. Horvathova, I. Hochova, J. Cermak, J. Blatny, M. Sukova, E. Bubanska, *et al.*, "Czech and slovak diamond-blackfan anemia (dba) registry update: Clinical data and novel causative genetic lesions," *Blood Cells, Molecules, and Diseases*, vol. 81, p. 102380, 2020.
31. S. S. Jin, C. J. Lin, X. F. Lin, J. Z. Zheng, and H. Q. Guan, "Silencing lncRNA NEAT1 reduces nonalcoholic fatty liver fat deposition by regulating the miR-139-5p/c-Jun/SREBP-1c pathway," *Ann Hepatol*, vol. 27, no. 2, p. 100584, 2022.
32. M. Zhou, Y. Sun, Y. Sun, W. Xu, Z. Zhang, H. Zhao, Z. Zhong, and J. Sun, "Comprehensive analysis of lncRNA expression profiles reveals a novel lncRNA signature to discriminate nonequivalent outcomes in patients with ovarian cancer," *Oncotarget*, vol. 7, pp. 32433–32448, May 2016.
33. G. R. Uhl and M. J. Martinez, "PTPRD: neurobiology, genetics, and initial pharmacology of a pleiotropic contributor to brain phenotypes," *Ann N Y Acad Sci*, vol. 1451, pp. 112–129, 09 2019.
34. E. Rothzerg, X. D. Ho, J. Xu, D. Wood, A. Märtson, and S. Kõks, "Upregulation of 15 antisense long non-coding rnas in osteosarcoma," *Genes*, vol. 12, no. 8, p. 1132, 2021.
35. W. Zhu, X. Xiao, and J. Chen, "Silencing of the long noncoding RNA LINC01132 alleviates the oncogenicity of epithelial ovarian cancer by regulating the microRNA-431-5p/SOX9 axis," *Int. J. Mol. Med.*, vol. 48, Aug. 2021.
36. Q. Sun, Y. J. Song, and K. V. Prasanth, "One locus with two roles: microrna-independent functions of microrna-host-gene locus-encoded long noncoding rnas," *Wiley Interdisciplinary Reviews: RNA*, vol. 12, no. 3, p. e1625, 2021.
37. Y. Wang, W. Li, X. Chen, Y. Li, P. Wen, and F. Xu, "MIR210HG predicts poor prognosis and functions as an oncogenic lncRNA in hepatocellular carcinoma," *Biomed Pharmacother*, vol. 111, pp. 1297–1301, Mar 2019.

38. G. Liu, L. Wang, and Y. Li, “Inhibition of lncRNA-UCA1 suppresses pituitary cancer cell growth and prolactin (PRL) secretion via attenuating glycolysis pathway,” *In Vitro Cell. Dev. Biol. Anim.*, vol. 56, pp. 642–649, Sept. 2020.
39. F. Peng, S. Yan, H. Liu, Z. Liu, F. Jiang, P. Cao, and R. Fu, “Roles of LINC01473 and CD74 in osteoblasts in multiple myeloma bone disease,” *J Investig Med*, Feb 2022.
40. W. J. Huang, X. P. Tian, S. X. Bi, S. R. Zhang, T. S. He, L. Y. Song, J. P. Yun, Z. G. Zhou, R. M. Yu, and M. Li, “The β-catenin/TCF-4-LINC01278-miR-1258-Smad2/3 axis promotes hepatocellular carcinoma metastasis,” *Oncogene*, vol. 39, pp. 4538–4550, 06 2020.
41. L. Jin, C. Luo, X. Wu, M. Li, S. Wu, and Y. Feng, “Lncrna-haglr motivates triple negative breast cancer progression by regulation of wnt2 via sponging mir-335-3p,” *Aging (Albany NY)*, vol. 13, no. 15, p. 19306, 2021.
42. J. Tang, J. Ren, Q. Cui, D. Zhang, D. Kong, X. Liao, M. Lu, Y. Gong, and G. Wu, “A prognostic 10-lncrna expression signature for predicting the risk of tumour recurrence in breast cancer patients,” *Journal of cellular and molecular medicine*, vol. 23, no. 10, pp. 6775–6784, 2019.
43. N. Dastmalchi, R. Safaralizadeh, S. Latifi-Navid, S. M. Banan Khojasteh, B. Mahmud Hussen, and S. Teimourian, “An updated review of the role of lncrnas and their contribution in various molecular subtypes of breast cancer,” *Expert Review of Molecular Diagnostics*, vol. 21, no. 10, pp. 1025–1036, 2021.
44. Q. Mao, M. Lv, L. Li, Y. Sun, S. Liu, Y. Shen, Z. Liu, and S. Luo, “Long intergenic noncoding RNA 00641 inhibits breast cancer cell proliferation, migration, and invasion by sponging miR-194-5p,” *J Cell Physiol*, vol. 235, pp. 2668–2675, 03 2020.
45. X. Han and S. Zhang, “Role of Long Non-Coding RNA LINC00641 in Cancer,” *Front Oncol*, vol. 11, p. 829137, 2021.
46. L. Jin, C. Li, T. Liu, and L. Wang, “A potential prognostic prediction model of colon adenocarcinoma with recurrence based on prognostic lncRNA signatures,” *Hum Genomics*, vol. 14, p. 24, 06 2020.
47. X.-y. Li, L.-y. Zhou, H. Luo, Q. Zhu, L. Zuo, G.-y. Liu, C. Feng, J.-y. Zhao, Y.-y. Zhang, and X. Li, “The long noncoding rna mir210hg promotes tumor metastasis by acting as a cerna of mir-1226-3p to regulate mucin-1c expression in invasive breast cancer,” *Aging (Albany NY)*, vol. 11, no. 15, p. 5646, 2019.
48. Y. Du, N. Wei, R. Ma, S.-H. Jiang, and D. Song, “Long noncoding rna mir210hg promotes the warburg effect and tumor growth by enhancing hif-1α translation in triple-negative breast cancer,” *Frontiers in oncology*, vol. 10, 2020.
49. W. Shi, Y. Tang, J. Lu, Y. Zhuang, and J. Wang, “MIR210HG promotes breast cancer progression by IGF2BP1 mediated m6A modification,” *Cell Biosci*, vol. 12, p. 38, Mar 2022.
50. J. Ma, F. F. Kong, D. Yang, H. Yang, C. Wang, R. Cong, and X. X. Ma, “lncRNA MIR210HG promotes the progression of endometrial cancer by sponging miR-337-3p/137 via the HMGA2-TGF-β/Wnt pathway,” *Mol Ther Nucleic Acids*, vol. 24, pp. 905–922, Jun 2021.
51. X. Li, F. Jin, and Y. Li, “A novel autophagy-related lncrna prognostic risk model for breast cancer,” *Journal of Cellular and Molecular Medicine*, vol. 25, no. 1, pp. 4–14, 2021.
52. X. Pan, D. Li, J. Huo, F. Kong, H. Yang, and X. Ma, “Linc01016 promotes the malignant phenotype of endometrial cancer cells by regulating the mir-302a-3p/mir-3130-3p/nfya/satb1 axis,” *Cell death & disease*, vol. 9, no. 3, pp. 1–18, 2018.
53. J. T. Hua, M. Ahmed, H. Guo, Y. Zhang, S. Chen, F. Soares, J. Lu, S. Zhou, M. Wang, H. Li, N. B. Larson, S. K. McDonnell, P. S. Patel, Y. Liang, C. Q. Yao, T. van der Kwast, M. Lupien, F. Y. Feng, A. Zoubeidi, M. S. Tsao, S. N. Thibodeau, P. C. Boutros, and H. H. He, “Risk SNP-Mediated Promoter-Enhancer Switching Drives Prostate Cancer through lncRNA PCAT19,” *Cell*, vol. 174, pp. 564–575, 07 2018.
54. N. Li and X. Zhan, “Identification of clinical trait–related lncrna and mrna biomarkers with weighted gene co-expression network analysis as useful tool for personalized medicine in ovarian cancer,” *EPMA Journal*, vol. 10, no. 3, pp. 273–290, 2019.

55. K. Dong, J. Shen, X. He, G. Hu, L. Wang, I. Osman, K. M. Bunting, R. Dixon-Melvin, Z. Zheng, H. Xin, M. Xiang, A. Vazdarjanova, D. J. R. Fulton, and J. Zhou, "Is an Evolutionarily Conserved Smooth Muscle Cell-Specific LncRNA That Maintains Contractile Phenotype by Binding Myocardin," *Circulation*, vol. 144, pp. 1856–1875, 12 2021.
56. S. Ghosal, B. Zhu, T.-T. Huynh, L. Meuter, A. Jha, S. Talvacchio, M. Knue, M. Patel, T. Prodanov, S. Das, *et al.*, "A long noncoding rna–microrna expression signature predicts metastatic signature in pheochromocytomas and paragangliomas," *Endocrine*, pp. 1–10, 2021.
57. Y. Zhang, X. You, S. Li, Q. Long, Y. Zhu, Z. Teng, and Y. Zeng, "Peripheral blood leukocyte rna-seq identifies a set of genes related to abnormal psychomotor behavior characteristics in patients with schizophrenia," *Medical science monitor: international medical journal of experimental and clinical research*, vol. 26, pp. e922426–1, 2020.
58. S. Li, J. Liu, F. Kong, Y. Wang, N. Li, and Y. Zou, "lncRNA GHET1 has effects in development of pre-eclampsia," *J Cell Biochem*, vol. 120, pp. 12647–12652, 08 2019.
