# Supplementary material for: MultiCens: Multilayer network centrality measures to uncover molecular mediators of tissue-tissue communication
Source: PLoS Comput Biol. 2023 Apr 24;19(4):e1011022. doi: 10.1371/journal.pcbi.1011022 (PMC10159362; doi:10.1371/journal.pcbi.1011022)
Supplement: Attachment — Submitted filename: v3.Reviewers Response Letter MultiCens.pdf [file pcbi.1011022.s002.pdf]

Dear Guest Editor Gregory W. Schwartz and Section Editor Mark Alber,

We thank you and the reviewers for providing us valuable feedback on the manuscript, including sharing the reviewers' appreciations of the manuscript as well as their concerns about certain analyses or sections of the manuscript.

We have diligently worked to address the reviewers' concerns in the revised manuscript. We have especially elaborated on the novelty and description of the method, and performed additional analyses related to both benchmarks and use-cases/applications. A summary of certain key analyses/revisions follows.

- In the revised text, we've now cited additional related works suggested by reviewers such as RWR-H (a representative method for a Random Walk with Restart based centrality for a Heterogenous network). In comparison to previous works which typically provide a single centrality score for each node in a heterogeneous/multilayer network, a novel aspect of our MultiCens framework is the multiple, hierarchically-related centrality measures we provide for each node. These MultiCens measures are well-founded due to the theoretical guarantees we prove about their decomposability and convergence.
- In additional comparative analyses, we highlight the similarities and advantages of MultiCens relative to a larger set of methods. Specifically, besides MultiCens query-set centrality, other MultiCens measures (local and global centrality) and existing methods (inter-layer degree, versatility, and RWR-H) have now been comparatively evaluated on synthetic benchmarks. Similarly, MultiCens query-set centrality and existing methods have now been evaluated for the query-set-based hormone-gene prediction task.
- Additional analyses have now shown the advantage of having multiple centrality measures per gene in the Alzheimer's disease (AD) application – different molecular pathways are revealed by different centrality-based rankings of the AD network genes.

Our point-by-point response to all reviewer concerns are provided below (in blue font), and it includes a detailed description of all additional analyses and text revisions we've done. The manuscript with the revisions highlighted in yellow color is also attached along with this rebuttal submission, and all page numbers indicated in this reviewer response document refer to page number in the revised manuscript (unless otherwise specified).

We are glad that the reviewers appreciate our work, and hope we've addressed their concerns in the revised manuscript and in this rebuttal document. We are open to make further revisions if necessary. We believe the manuscript has strengthened much from incorporating the reviewers' inputs, and we look forward to hearing from you.

Thank you,
Manikandan Narayanan
(on behalf of all co-authors)

****

<u>Reviewer 1:</u>

This paper presents a new set of PageRank-style metrics representing different types of node centrality in multilayer networks. As opposed to the previously published "versatility" measure, which simply computes PageRank statistics on tensors, here the authors define separate local centrality (within-layer) and global centrality (between-layer connections not captured by local centrality) metrics. If the global centrality is then defined relative to a specific set of target nodes and propagated within each layer via local-set centralities, this is defined as "query-set centrality." The authors show that the query-set centrality is superior to versatility and inter-layer degree to identify important nodes in simulated multilayer networks. They apply query-set centrality to tissue-specific networks from GTEx and BioSNAP to identify the connections between hormone-producing and hormone-responsive genes. They also present an application to data from different brain tissue types in Alzheimer's patients.

Overall, the paper is logically presented, and the analyses are clearly described. Code is provided on GitHub. The results would be of interest to the community, if further clarification is added to the paper.

We thank the reviewer for insightful and positive feedback. We have tried our best to answer all of your questions below.

<u>Comments-</u>

Q1: The paper says MultiCens is different from versatility and other methods "due to its ability to distinguish intra- vs. inter-layer edges." In versatility, the random walk goes from a node to any neighboring node in the same layer or different layer. One could imagine scaling the inter-layer edge weights to change the rate of hopping through inter-layer edges, so in that sense, inter- and intra-layer edges are distinguishable. Why is versatility not able to identify source set 2 as being connected to the query set in Figure 2, when versatility allows hops both between and within layers of the network? Can you explain more about what causes the improvement in query-set centrality? Is it the use of local-set centrality in weighting intra-layer hops? Further explanation would help the reader grasp the innovation in this method.

Response:

Thank you very much for your thoughtful suggestion. One can assign a higher weight to the inter-layer edges to increase the ranking of nodes with more across-layer edges. This form of re-weighting would be applied to connections leading to the entire layer though, not a specific set of nodes (query nodes) in the target layer. So versatility fails to capture *source set 2* more due to its inability to focus on the query nodes (it captures influence of a node on all other nodes); and less due to its inability to distinguish intra- vs. inter-layer edges. We have revised the text in the Results section on synthetic benchmarks to clarify this point.

In general, query-set centrality gains from within-layer and across-layer (single/multi-hop) connectivity focused towards the query-set, and from the bias introduced by the intra-layer local-set centrality (also towards the query-set). In the synthetic multilayer network, *source set 2* gets a high rank because of its within-layer connections to *source set 1*, which in turn gets high centrality because of its direct across-layer connections to the *query set*. Local set centrality can also help propagate influence towards query nodes via within-layer links, and this bias can be useful when the query nodes are not well connected via inter-layer connections.

Q2: Minor question related to the previous comment. The query-set centrality explicitly needs the query set of nodes as input to the metric. In Figure 2, how is the query set used as input when computing the versatility and the inter-layer degree?

Response:
Thank you for your question. Versatility and inter-layer degree methods inherently don't consume a target set of nodes. We have done experiments with these two settings:

1. For a fair comparison with our query-set centrality method, we modify the multilayer network structure by preserving all edges in the first layer and only those edges in the 2nd layer that involve nodes from the query set. It means the query set in the 2nd layer will have both within-set links, and those across-set links that connect to the first layer.
2. Use the multilayer network structure without any modifications.

In both settings, versatility and inter-layer degree are unable to recover the ground truth nodes from the *source set 2*. Versatility ranks other communities (3 communities marked by red color in Fig. 2) better, whereas inter-layer degree always ranks *source set 1* better.

In the paper, we report plots obtained using the first setting (Fig. 3 in main text and Fig. S2 in supplement).

Q3: The query-set centrality appears to perform well in simulations. However, it is difficult to know whether the simulation mimics the real biological situation. Can the authors compare the performance of MultiCens with the other methods (versatility and inter-layer degree, with query set as input) when applied to the tissue-specific GTEx and PPI networks? This will demonstrate if multi-hop paths are important for connecting hormone-responsive and hormone-generating genes.

Response:
Thank you for providing these inputs. We've now added recall-at-k plots in Fig. S2 to compare different centrality measures on the hormone-gene prediction task (done using the GTEx/PPI-based networks), and summarized key findings in the section "MultiCens ranks

inter-tissue signaling genes at the top". Different methods offer unique insights into the biological system, with no one measure being universally effective. Overall, MultiCens query-set centrality (QC) performs better than or comparably to other methods with some exceptions like when predicting progesterone-responding genes.

Q4: There is a section on lncRNAs and their importance in tissue-tissue communication. Are lncRNAs found to be more connected to hormone-activating/responding genes than randomly chosen genes? Can the authors provide a p-value to show that lncRNAs are particularly important in this context?

Response:
We are sorry for this misunderstanding. We did not mean to claim that lncRNAs are more important than protein-coding genes. The top-ranked lncRNAs are biologically significant as described in the main text and hence we thought it would be worth mentioning it, as the role of lncRNAs in hormone regulation is often overlooked. As suggested by the reviewer, we tested the importance of lncRNA genes with that of random genes (including protein-coding genes) using insulin hormone as the test case.

In order to check if the lncRNAs in the pancreas tissue are connected to insulin-responding genes with more strength than a random set of genes in the pancreas, we performed a stratified random sampling of 100 sets of genes, each being the same size as the set of lncRNAs, and the strata/level being defined using the variance of gene expression levels. Our results indicate that lncRNAs are less connected than randomly selected genes with matching size and gene expression variance levels. Despite lack of statistical significance, our literature-based associations (in Table 1, Table S3 and the accompanying text) reveal that the top-ranked lncRNAs have biological significance for the hormone being studied. Fig. R1 below illustrates the distribution of ranks (lower the better) for lncRNAs (left) and randomly selected genes (right) according to MultiCens QC.

We have mentioned this point in the Discussion, and hope the role of lncRNAs in hormone regulation will be studied thoroughly in the near future.

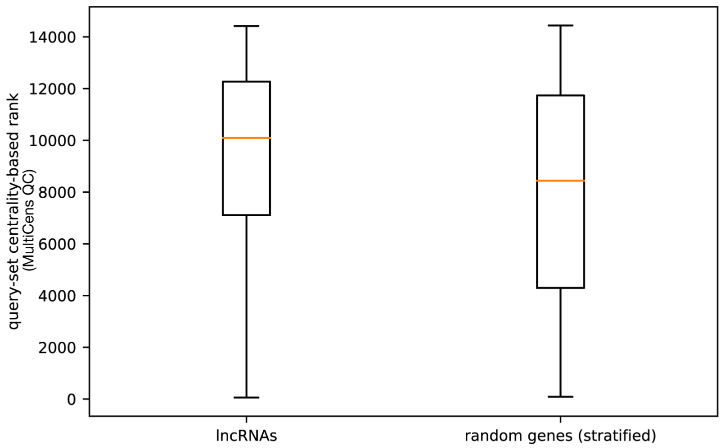

Fig. R1: *MultiCens QC ranks of the set of lncRNAs vs. a set of random genes of the same size.*

****

<u>Reviewer 2:</u> Kumar and colleagues present a novel approach to identify key genes in multi-organ gene co-expression networks. Their methodology include a set of novel centrality measures, developed with a strong mathematical fundamentation; their usefullness is demonstrated in a well-organized set of examples. Overall, it is a fine study and I can see the utility of their methods in research questions outside their original application.
At the same time, after a careful evaluation of their study, there are a few points that require a better explanation, since they are key to the demonstration of the real validity of their methods, as well as its ability to really uncover meaningful relations.

We thank the reviewer for comments on the utility of our work to the scientific community. We hope our edits and responses below satisfactorily address all the concerns the reviewer has noted.

<u>Comments-</u>

Q1: Gene expression databases have a large variability: GTEx was the base of their study - its gene expression values were derived from a large set of human subjects. It is well known that this dataset has a large internal variability – namely, the expression of single genes have a broad range of values, reflecting physiological, sex and age related differences. In this sense, it is essential for the authors to take this variability into account, otherwise they are using the mean expression of genes – a profile of an individual that does not exist. There are multiple ways to do that, e.g., using other databases like HPA RNA Seq, Illumina's body2Map, or BioGPS. Alternatively, they can use GTEx itself, by perturbing the gene expression values within the boundaries of their expression ranges.

Response:
We agree with you that the individual-to-individual variability in the GTEx data needs to be taken into account. That variability in the GTEx data is precisely what we exploit to build our correlation networks among genes within and across tissues. If two genes exhibit co-variability (correlation) across all individuals sampled in the GTEx data, we take that as an indication of a functional association between the genes.

But the above premise relies on the variability in the GTEx data to be based mostly on biological factors, rather than confounding technical factors. Towards this end, please note that we've adjusted the GTEx data for certain covariates that could potentially confound the gene-gene coexpression relations, including gender, PEER factors (which are latent data-driven variables that capture unknown confounding factors), sequencing platform, genotype PCs (principal components of the genetic data that capture underlying population substructure among the GTEx individuals), etc. So the variability explained by these potential confounding factors

are accounted for and removed using a linear regression model before building the coexpression networks. A clarification regarding this has been added to the "Real-world Application I" section in Methods.

Furthermore, please note that we don't work with atlas data or mean expression of genes across a set of individuals, but instead work with expression data collected from hundreds of individuals (in the GTEx data indeed), and estimate the correlation coefficient between every pair of genes across these individuals. These correlations are then collated into a gene-gene coexpression network.

Q2: Protein and mRNA levels: While I understand that this approach is based on a gene co-expression network, readers will immediately wonder how these findings are reflected in the protein world – because ultimately, the proteins are the entities that make things happen in the organism. The authors should address this issue, using for example the Protein atlas database.

Response:
Thank you very much for the suggestion. In the current manuscript, we focused on gene-gene coexpression networks due to availability of large cohorts of data (e.g., GTEx and MSBB AD data, where several hundreds of individuals were expression-profiled). It will also be interesting to apply MultiCens to multi-tissue proteomics data that become available on large cohorts of individuals - we've mentioned this in Discussion as a future work.

Please note that this study focuses on correlation between two genes (or proteins) across a set of individuals, and not across a set of spatial regions/sites in a gene (or protein) atlas. So as such, we need large cohorts of individuals in which multiple proteins are measured to build reliable protein coexpression networks. Atlas data are obtained across several spatial locations but typically from only a few individuals/replicates, and hence are not a good match for building the coexpression networks focused in this study.

Q3: Random networks: I would like to see how their novel centrality measures behave in random networks; there are two ways to do that, (i) simply shuffling the gene labels, in a way not to alter the node distribution and inner network structure; (ii) shuffling all edges, to purposefully modify the network structure. In principle, I would expect that their centrality measures do not uncover any meaningful relationships in these networks.

Response:
We thank the reviewer for this valuable suggestion and agree that it would strengthen our current study. We have implemented the reviewer's random network scheme (i) of shuffling gene labels and added it as a new functionality to our MultiCens software. We have also demonstrated this functionality in Suppl Fig. S1 (see also Fig. 4 in main text) by showing that the centrality scores

of ground-truth genes (either insulin-producing or insulin-responsive genes) are better in the actual network than the gene-labels-shuffled network. This is equivalent to showing that the centrality scores of the ground-truth set in the network are better than that of size-matched random gene sets.

We also added a refinement to the above procedure. Instead of considering a random gene set that matches only the size of the ground-truth gene set, we choose a random set that also matches other properties of the ground-truth set to get a more stringent null model. Our implementation stratifies genes by their expression variance across all sampled individuals into three strata (low, medium and high variance), and then matches the proportion of these three types of genes between the random set and the ground-truth set. With this refined shuffling procedure, we observe the same trend as above (see Suppl Fig. S1), i.e., we do not uncover any meaningful relationship in this refined random network as well.

Above discussion has been added as a new Methods section titled "Centrality of random node sets to assess statistical significance". Please note that we did consider your scheme (ii) for shuffling the gene network, but our network is a fully-connected (complete) weighted graph, and there was no consensus in the literature on how to properly shuffle/rewire weighted edges of a graph. So we focused on scheme (i) and its refinement, as discussed above.

Q4: Arbitrary values: The authors selected arbitrary cutoff values without a statistical or biological justification. For instance, on page 6, the authors chose to inspect further the subnetworks with at least 10 genes on the producing and responding tissues. On page 23, line 660, the authors selected 10,000 genes as their limit of genes per tissue. On page 25, the authors selected the top 9,000 most varying genes. Without a proper justification, it seems that the authors selected these values only because they work well with their methods.

Response:
We understand your concern and appreciate pointing that out.

Regarding the 10-gene cutoff for the hormone-gene prediction task, we had already presented results for ground-truth gene sets with fewer than 10 genes in Suppl Fig. S2 in the original manuscript (now Suppl Fig. S3 in the revised manuscript) for all tested hormones. You can observe from this figure that the recall-at-k curves are very step-like or non-smooth for ground-truth set sizes of 1-4 or 7-8, and hence we cannot make robust conclusions about relative performance of different methods from the area under such curves. So we decided to focus on a size-10 cutoff for getting reliable results that are shown in the main text. The readers can refer to the recall-at-k curves in Suppl Fig. S2 to see the results for all tested hormones.

Regarding the size of genes in the overall network, please note that we apply MultiCens on fully-connected (complete) weighted graphs, and the supra-adjacency matrix of such graphs would be cumbersome to learn and analyze for a large number of nodes. To avoid this blow-up in the size of the multilayer network, we only use the top 10,000 varying genes in each tissue and take the union of these genes while constructing the multilayer network. However, with 9,000 genes, we got similar results (see Fig. R2 below) as those with 10,000 (see Suppl Fig. S2, top panel).

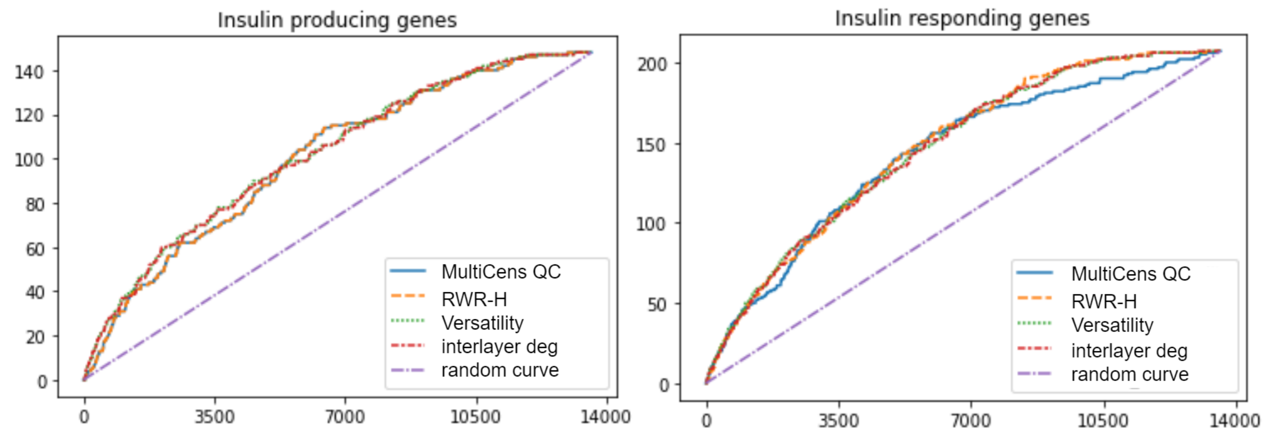

Fig. R2: *Recall (number of ground truth genes recovered; y-axis) in the top k ranked genes (x-axis) are plotted using MultiCens query-set centrality (QC) based ranking vis-à-vis rankings obtained by baseline methods. Here, the union of 9000 most-varying genes in each tissue is taken as the node set of the multilayer network.*

Having shown that both 9,000 and 10,000 genes perform similarly for the hormone-gene application, we note that we couldn't try 10,000 genes for the AD (Alzheimer's Disease) application of MultiCens for the following reason. Since there are 2 more layers in the multilayer network in the AD application than for the hormone-gene application (i.e., 4 vs. 2 layers respectively for AD vs. hormone-gene application), taking the union of 10,000 most-varying genes across four brain regions resulted in prohibitive running time for building and analyzing the multilayer network. So, a smaller cutoff of 9,000 was chosen for AD application and 10,000 for hormone-gene application.

Q5: Traditional centrality measures: I would like to see how the "classical" network centrality measures perform in comparison to the measures that the authors introduced. For example, the betweenness, closeness and others can take into account the weight of edges as well as directionality, if necessary.

Response:

We thank the reviewer for this valuable suggestion and agree that it would be interesting to carry out this study. However, in this case, it is outside the scope of the paper because centrality measures such as betweenness or closeness need to be first extended to multilayer networks and then compared with existing measures. Such extensions will definitely bring out interesting insights - for example, betweenness centrality measure can reveal interesting intermediary genes for a given source and target set of genes. Our problem setting in this work is different, as we want to discover the source set of a hormone (such as insulin-producing genes) for a given set of responsive genes in another tissue (such as insulin-responding genes).

****

Reviewer 3: In their manuscript, Kumar and colleagues introduce a multilayer network prioritization method that they devised, called MultiCens, to identify important, or "central" nodes in a multilayer network. They focus on a particular theme, namely inter-tissue communication networks (ICN), and devote the majority of the main text to the demonstration of their method on several use cases in that vein, e.g., hormone-receptor relationships across tissues. ICN seems to be a relatively recent research area, and the use of multi-tissue genomic datasets in the context of multilayer networks is potentially useful. However, the authors state in the Introduction that the main contribution of their work is the design of a new multilayer centrality measure, which, in my view, is not sufficiently supported in the paper. Below are my major concerns about the paper relating to this point and some others:

We appreciate the reviewer's effort and time in evaluating our manuscript. We have incorporated changes that reflect the detailed suggestions you have provided.

Comments-

Q1: Multilayer networks have been thoroughly investigated in the past decade; therefore, the claim to a novel multilayer centrality measure has to be properly justified, which is currently lacking in the paper. In particular, the authors seem to be unaware of a considerable body of previous or recent works that are very similar to their "multi-hop" approach, i.e., diffusion and random-walk based methods, some of which are included below:

• [Most notably] Valdeolivas, Alberto, et al. "Random walk with restart on multiplex and heterogeneous biological networks." Bioinformatics 35.3 (2019): 497-505.
• Baptista, Anthony, Aitor Gonzalez, and Anaïs Baudot. "Universal multilayer network exploration by random walk with restart." Communications Physics 5.1 (2022): 1-9.
• Bergermann, Kai, and Martin Stoll. "Fast computation of matrix function-based centrality measures for layer-coupled multiplex networks." Physical Review E 105.3 (2022): 034305.
• Bergermann, Kai, and Martin Stoll. "Orientations and matrix function-based centralities in multiplex network analysis of urban public transport." Applied Network Science 6.1 (2021): 1-33.

More examples that don't claim method novelty but are novel applications, like the present paper, can also be found. Some examples below:

• Qu, Jia, et al. "Biased Random Walk With Restart on Multilayer Heterogeneous Networks for MiRNA–Disease Association Prediction." Frontiers in Genetics (2021): 1427.
• Tang, Yujiao, et al. "DRUM: inference of disease-associated m6A RNA methylation sites from a multi-layer heterogeneous network." Frontiers in genetics 10 (2019): 266.

Finally, the multilayer version of pagerank itself is not new -- it even predates what is called the seminal contribution in the paper:

• Halu, A., Mondragón, R. J., Panzarasa, P., & Bianconi, G. (2013). Multiplex pagerank. PloS one, 8(10), e78293.

Response:
We thank the reviewer for pointing out these related works - we have now added them to the manuscript, and clarified the novelty of our work in light of these existing works upfront in the beginning of the manuscript (specifically in Page 3 of the revised manuscript), and also in the end of the manuscript (last paragraph in Discussion of the revised manuscript).

While we had already cited several multilayer network studies, we apologize for missing these specific related works pointed out by the reviewer. Some of the missed works pertain to centrality of ***multiplex*** networks, which are a popular yet restricted class of multilayer networks wherein the only inter-layer edges allowed are "identity" edges between the same nodes in different layers. For instance, the centrality by Halu et al. (2013) belongs to this special class of networks. On the other hand, the "versatility" centrality by Domenico et al. (2015) is expressed using a general multilayer network representation (using the notation of a 4-rank adjacency tensor that can represent intra/inter-layer edges between any two nodes in the overall network), and is therefore closer to our work and cited as such.

More recent works by Valdeolivas et al. (2019) and Qu et al. (2021) pointed out by the reviewer present centrality for nodes in a ***heterogeneous*** network model, which can be thought of as equivalent to a multilayer network model (as explained in *Background and Preliminaries* section in Methods). The centrality measures proposed in these recent studies are mostly based on the idea of RWR-H, random walk with restart from a set of seed nodes in a Heterogeneous network (with this RWR-H idea introduced in an earlier work by Li and Patra (2010) [reference #30 in the revised manuscript]). Our MultiCens framework has a unique advantage over these existing methods in its ability to decompose the centrality of a node into contributions from local vs. global effects (specifically, the local effect of nodes within a layer vs. the global effect of nodes in different layers). In detail, we offer several hierarchically-related MultiCens centrality measures based on local vs. global vs. layer-specific vs. query-set centrality, whereas the earlier RWR-based methods provide only one centrality measure for each node in a heterogeneous network.

To summarize, the key novel aspects of our proposed MultiCens centrality measures are:

1. Compared to earlier studies on versatility which doesn't distinguish between intra- vs. inter-layer edges, and S_{sec} which captures only single-hop interactions, MultiCens can exploit the multi-layer, multi-hop connectivity structure in its centrality measures.
2. Compared to earlier studies on the centrality of a ***multiplex*** network, our MultiCens measures work for the general class of multilayer networks (which includes multiplex networks as a restricted sub-class).

3. Compared to earlier studies on RWR-based centrality of a *heterogeneous* network, we provide different types of MultiCens measures, which can delineate the centrality of a node at local intra-layer vs. global inter-layer vs. other hierarchically decomposable levels in a theoretically well-founded fashion. This quantification of local vs. global centrality is important for our multi-tissue systems biology setting, in order to understand whether a gene exerts its influence on other genes via within-tissue molecular pathways, or via across-tissue communication routes, or via a combination of both.

Please refer to Fig. 3, Suppl Fig. S2, and Suppl Figs. S5-S6 and their associated text for more discussion of the need and novelty of the multiple types of multi-layer network centrality measures, MultiCens, proposed in our work.

Q2: In light of the above, the benchmarking, in its present form, is lacking key comparisons. The authors need to compare their method to more of the methods that are much more similar to theirs, such as the above. Of course, some of these works are quite new and we can't expect a benchmark that includes all of these approaches; but the authors must, at minimum, include some of the more established methods above (such as RWR-MH) and demonstrate the advantage of MultiCens over them. Related to this point, the authors actually only compare their method with other methods in the synthetic case (Figure 2). Figure 3 has no comparison with any other methods, not even with single layer methods such as versatility. As such, the AUC values don't have much meaning, e.g., 0.6 vs 0.7, other than that they're better than random expectation, which isn't a high bar for a new method (and this is the case for only 3/4 cases). The methods included for Fig 2. must therefore be in Fig. 3 as well, in addition to the further benchmark request above.

Response: Thanks for this suggestion - we have now added the key comparisons you've mentioned. Besides MultiCens query-set centrality (QC), other MultiCens measures (local and global centrality), and three existing methods (inter-layer degree, versatility, and RWR-H) have now been comparatively evaluated on synthetic benchmarks (Fig. 3 of revised manuscript). Similarly, MultiCens QC and the three existing methods have now been evaluated on the query-set-focused hormone-gene prediction application (Fig. S2). In these additional comparative analyses, we highlight the similarities as well as advantages of our MultiCens measures in comparison to other methods like RWR-H.`

Note that we chose RWR-H as the representative state-of-the-art method to compare MultiCens against for the following reason. Previous works on centrality for heterogeneous/multilayer networks, and not multiplex networks, are the most relevant methods to compare MultiCens against (see also response to the last question). All four recently published heterogeneous/multilayer network centrality methods [Valdeolivas et al. (2019), Qu et al. (2021), Baptista et al. (2022), Tang et al. (2019)] were based on the concept of Random Walk with

Restart from a seed set of nodes in a heterogeneous/multilayer network, and all of these studies are also adaptations/extensions of an earlier study by Li and Patra (2010) [reference #30 in the revised manuscript]. In fact, for the case of two-layer networks such as in our synthetic benchmarks and hormone-gene use-cases, all of these studies except Qu et al. (2021) will result in the same centrality definition, which we refer to as RWR-H. The other method in Qu et al. (2021) is based on a degree-biased random walk with restart, and this BRWR-H method performed comparable to or worse than RWR-H in predicting hormone-gene relations (see Fig. R3 below, side-by-side with Suppl Fig. S2), and so we present only RWR-H results in the revised manuscript for simplicity.

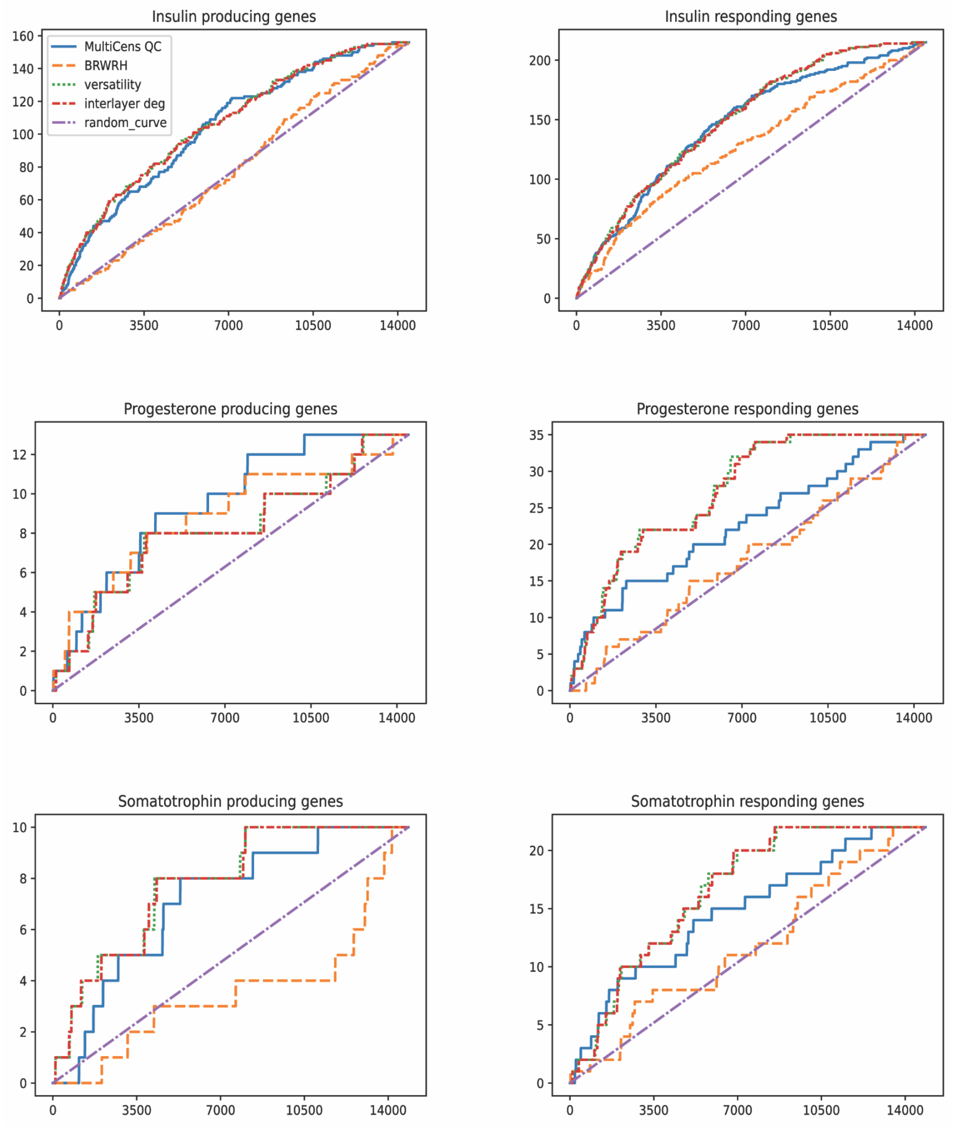

Fig. R3: *Recall (number of ground truth genes recovered; y-axis) in the top k ranked genes (x-axis) are plotted using MultiCens query-set centrality (MultiCens QC) based ranking vis-‘a-vis rankings obtained by baseline methods.*

Q3: "Despite the focus on the novelty of the method, the method itself is not sufficiently described in the main text. A minimum amount of sufficient information must be present in the main text to understand the method. The authors seem to have focused on describing the use cases, which is fine, but the reader is left wondering what the method does exactly and how it works and how this centrality translates, intuitively, to the context of ICN. The only part I was able to clearly understand is that it somehow involves a source and a target (query) tissue. Questions that remain, without having to delve into the methods, are: How are interlayer connections defined? What are gene-gene interactions? Are the networks heterogeneous networks, or multiplex networks? What are "communities" in the synthetic case? Are these really network communities? Are they found through multilayer community detection methods? What does "adding communities on top of the multilayer network" mean? How are the two communities selected? What does a node becoming part of the ground truth mean? How is connection strength defined?
I don't mean to overwhelm the authors with questions – these are just some examples of what confused me as the reviewer, as there seems to be disconnect between the short method overview section and the synthetic use case. Please expand the former so that the results can be interpreted better."

Response:
We thank the reviewer for conveying the difficulty with the current organization of the manuscript. We have reorganized the manuscript in such a way that the "Materials and Methods" section precedes the "Results" section now. We believe this would address all the reviewer questions and concerns raised here, and should provide all methods' description beforehand to aid in the interpretation of the results.

We also request the Editor to permit this revised organization. We had initially followed the standard organization of a PLoS Computational Biology manuscript, wherein the "Materials and Methods" section follows the "Results" and "Discussion" sections; but in light of the reviewer suggestions and our response above, we request the Editor to permit "Materials and Methods" to precede the "Results" section as in the revised manuscript.

Q4: Potential confounding for enrichment analysis: The predictions for the hormones are made on their relevant tissue e.g., pancreas for insulin. The GTEx and SNAP networks for these tissues may already be enriched for tissue-specific diseases such as T2D regardless of the multicen ranking, as these networks contain a tissue specific subset of genes. The authors should accompany these findings with results showing that, e.g., T2D is not enriched in randomly ranked genes in the pancreas tissue.

Response:
We are sorry that this part was not clear in the manuscript. We use the WebGestalt web portal to identify enriched pathways. We input the gene symbols and their query-set centrality scores into the portal and set a threshold of 0.05 for the false discovery rate (FDR). Only the results that meet this threshold are reported. The WebGestalt portal uses 1000 permutations (random rankings) and the original query-set centrality based ranking in its Gene Set Enrichment Analysis (GSEA) method, and applies the Benjamini-Hochberg procedure to calculate the FDR for disease set enrichment. Therefore, the enrichment results provided in the manuscript, including those related to Type 2 Diabetes (T2D), are statistically significant.

Q5: Top 10 seems too restrictive for the PubMed query analyses. One would expect that the usefulness of a new method extends beyond its top 10 predictions out of 1000s of genes. How does the performance look like for top 100?

Response:

Thank you for your question. We have described the top 10 genes in the main manuscript because we did not want to overwhelm the readers with too many genes and their corresponding description. Further, in this case we have restricted our search among genes involved in peptide secretion, so we are limited with our search. There are only a few hundreds of secretory genes.

For understanding the signal in more numbers of genes such as top 100s or top 1000s, we have already presented other analyses such as recall-at-k analysis in Fig. 4, and enrichment analysis in Fig. 5a. The PubMed query analysis in Fig. 5b is supposed to complement these other analyses by inspecting a smaller set of genes at greater depth.

Q6: In Fig5, is the centrality here the proposed new centrality measure? Can these changes be explained partly by differences in topology e.g. differences between degree distributions of the AD vs ctrl networks? What do the box plots look like in terms of simple centrality measures such as degree, betweenness, etc.?

Response:
Thank you very much for the suggestion. Yes, plots in Fig. 5 (currently Fig. 6 in the revised manuscript) were obtained using the query-set centrality measure MultiCens QC. We have updated the text and figure legend accordingly, to make it more clear.

To understand the difference between the topology of the AD vs. CTL networks, and to obtain boxplots for a degree-based measure, we checked the distribution of the inter-layer degree of genes in FP, STG and IFG regions to the query-set of synaptic signaling genes (SSG) in PHG and obtained the following box plots (Fig. R4). Comparing these box plots with Fig. 6a in the

manuscript, it is clear that the order of regions is not exactly the same between different centrality schemes.

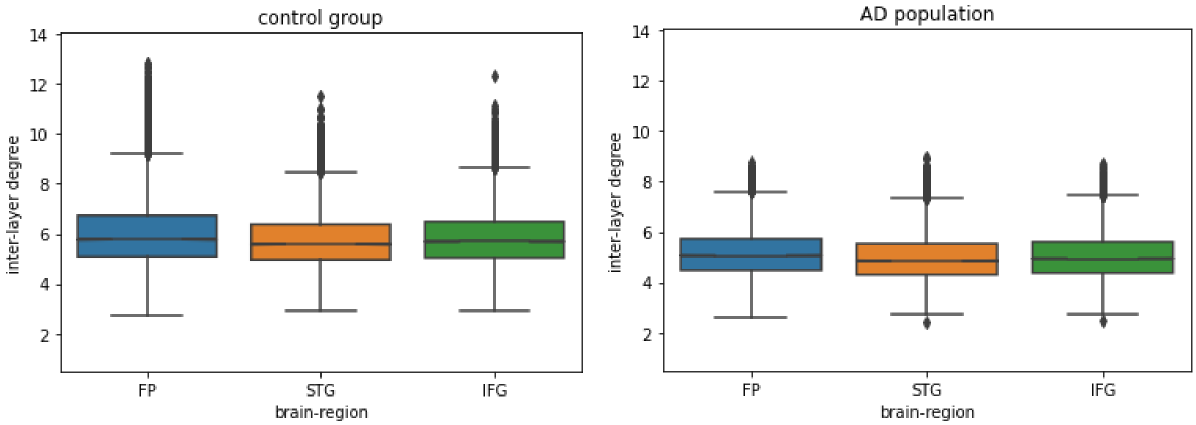

Fig. R4: Box plots showing region-wise shift of centrality scores of the three regions.

Further, we analyze the relation between QC-based ranks and interlayer degree-based ranks of the AD vs. CTL networks, and observed that across a large number of genes, the difference in ranks (AD rank - CTL rank) are correlated to a weak extent between both ranking schemes (Fig. R5 below). This reveals that the shift (difference) in the ranking of genes between AD vs. CTL network is not just based on the direct connections between genes (degree), but also based on multi-hop neighbors (as captured by MultiCens QC). This shows the advantage of our measures like MultiCens QC in a real-world disease genomics application.

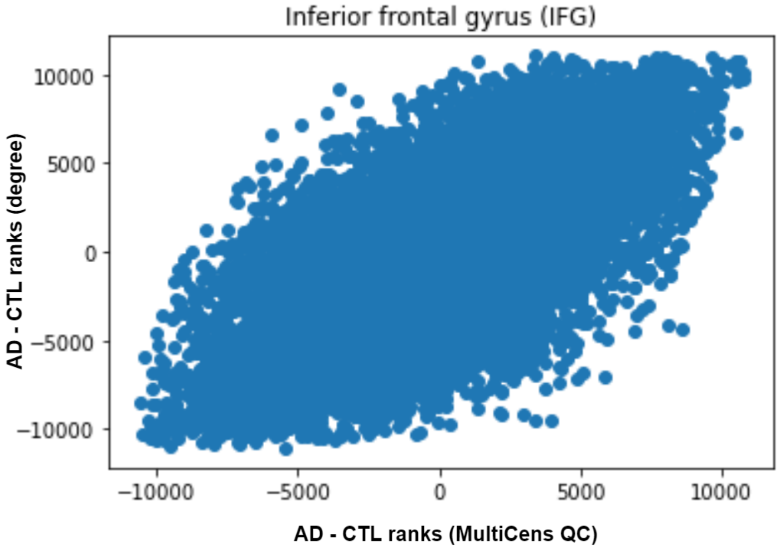

Fig. R5: *Scatter-plot representing the difference between MultiCens QC-based ranks and interlayer degree-based ranks of the AD vs. CTL network*

As a final note, we focused on degree-based and not betweenness centrality measures for the following reason. Betweenness measures can reveal interesting intermediary genes for a given source and target set of genes, but we've a different problem setting where we try to discover the source set (e.g., insulin-producing genes, or genes that interact with SSG) for a given target set in another tissue (e.g., insulin-responding genes, or SSG).

Q7: All main results seem to be derived from the query-set centrality; however, the first figure implies (as well as throughout the text) that MultiCens consists of "a set of" different hierarchical measures. What is the relation of these to MultiCens? Where are local, global, and layer-specific parts of MultiCens used or discussed? How are these relevant?

Response:
Thank you very much for bringing up this point. Originally, we have highlighted results that are derived from the query-set centrality. However, as the reviewer mentioned, MultiCens can be used to calculate different centrality measures including Query-set (QC), Local (LC) and Global (GC), and this set of different well-founded decomposable measures is in fact a main novel contribution of our study. While in Fig. 6a, we have previously presented only QC measures, we have now computed the local and global centrality measures for the genes involved in SSG (synaptic signaling genes) and have included a comparison across the three types of measures in the main text (both in the Methods and Results section on AD) and included the corresponding figures and tables in supplement (Suppl Tables S4 & S5, Suppl Figs. S5 & S6).

Our analysis clearly shows the difference among the three measures, and points out the different facets of the disease network (in terms of the different enriched molecular pathways) that the MultiCens measures uncover. Different centrality measures give different ranks to the genes, which through enrichment analysis (Gene Set Enrichment Analysis, GSEA using Webgestalt) result in different biological processes (Gene Ontology Biological Process, GO_BP; and pathways (Reactome)). This analysis shows that whereas certain processes like RNA splicing are important for within-brain-region function, certain others play a more influential role in across-region connectivity (please see pages 17 & 25 in the main manuscript and pages 7-10 & 16 in the supplementary document for a description of these results).

Minor issues:

1) All the information in Fig 3a seems to contained in fig 3b? Is 3a redundant then?

Response:

This is our current Fig. 4. Fig. 4a shows the trend of recall-at-k curves. Many times the AUC scores alone (Fig. 4b) can be misleading as the same AUC score can have different recall curve patterns. So we provide Fig. 4a to show the recall-at-k curves for our primary hormones and Suppl Fig. S3 for the remaining tested hormones. To understand the performance trend with respect to the size of ground truth genes, Fig. 4b is useful.

2) Findings on non-coding RNAs are potentially interesting, especially given that the function of long ncRNAs are still largely unknown. But then again, the results are mostly descriptive and need further validation. Since there is no sufficient ground truth information on these, the authors can just note this as a limitation in the discussion.

Response:
We found biologically significant connections between lncRNAs and hormone related genes, as described in the main text. Hence, we thought it is worth mentioning it, as the role of lncRNAs in hormone regulation is often overlooked. We agree with you that no ground truth data is available in the case of lncRNAs. We have mentioned this point in the discussion, and hope the role of lncRNAs in hormone regulation will be studied thoroughly in the near future.

3) Versatility is not a type of centrality but an alternative to it. There are versatility analogs of centrality measures, such as eigenvector and pagerank versatility.

Response:
Thank you for pointing this out. In the manuscript, we meant pagerank versatility. We have added a note in the manuscript in the Methods section (in *Background and Preliminaries*).

4) P13 line 326 typo "would've difficulty""

Response:
We have fixed it. Thank you for pointing it out.
